# Predicting condition-aware drug-induced transcriptional responses via a latent diffusion model

**DOI:** 10.1093/bioinformatics/btag173

**Published:** 2026-04-08

**Authors:** Chaewon Kim, Sunyong Yoo

**Affiliations:** Department of Intelligent Electronics and Computer Engineering, Chonnam National University, Gwangju, Republic of Korea; Department of Intelligent Electronics and Computer Engineering, Chonnam National University, Gwangju, Republic of Korea; R&D Center, MATILO AI Inc, Gwangju, Republic of Korea

## Abstract

**Motivation:**

Accurate prediction of condition-aware drug-induced transcriptional responses is essential for drug discovery and precision medicine. Current computational models, including encoder–decoder architectures and generative adversarial network-based approaches, exhibit reasonable performance but frequently neglect biological characteristics and fail to generalize to unseen conditions. Thus, this study presents a latent diffusion model that combines a variational autoencoder (VAE) with a diffusion process.

**Results:**

The VAE compresses gene expression (GE) profiles into a low-dimensional latent space, where the diffusion process learns the joint probability distribution over latent GE representations and noisy intermediates, thereby enabling more effective capture of gene–gene correlations. The model incorporates multiple perturbation conditions, including cell line, compound, dose, and time, to enhance prediction performance on unseen conditions. The reverse diffusion process predicts both the mean and variance of the posterior distribution, improving the fidelity of the generated GE profiles. The proposed model achieved the highest reconstruction performance in the unseen compound split with a Pearson correlation coefficient of 0.870 ± 0.001 and an *R*^2^ score of 0.739 ± 0.001, outperforming previous approaches. The model demonstrated superior preservation of gene–gene correlations, as confirmed by heatmap analysis. To evaluate biological relevance, we predicted half-maximal inhibitory concentration using generated GE, outperforming baseline methods. Latent space analysis revealed that the model preserved cell line identity and continuous dose–time variation. Gene set enrichment analysis confirmed that predicted GE reproduced known pathway-level responses to perturbation. These results demonstrate diffusion-based generative models as effective tools for modeling transcriptional responses in drug discovery and precision medicine.

**Availability and implementation:**

Source code and dataset are available at https://doi.org/10.5281/zenodo.18871024.

## 1 Introduction

Drug-induced cellular response refers to the physiological and biochemical changes that cells undergo in reaction to external signals, such as drug treatment (Herwig 2014). These cellular responses provide insights into disease treatment, drug development, and precision medicine (Ma *et al.* 2006, Zhao *et al.* 2014). Accurate prediction of cellular responses can help reduce the high costs associated with drug development and predict potential adverse effects in advance.

Transcriptome-level changes have been utilized as representative metrics to characterize cellular states and the associated directional changes (Pham *et al.* 2021, Szalai and Veres 2023). Gene expression (GE) patterns at the transcriptomic level reveal how drugs activate or inhibit biological pathways, enabling quantitative analysis of drug-induced cellular responses and gene−gene interactions (Herwig 2014, Pak *et al.* 2023, Szalai and Veres 2023, Bang *et al.* 2024). Recent advances in high-throughput screening technology have enabled rapid acquisition of large-scale transcriptomic data, promoting the establishment of comprehensive databases, such as the Library of Integrated Network-Based Cellular Signatures (LINCS) L1000 project (Pereira and Williams 2007, Subramanian *et al.* 2017). However, obtaining transcriptomic data for a large number of drug–cell combinations is experimentally impractical (Zhao *et al.* 2014). Thus, efforts have increased to predict drug-perturbed GE using computational methods.

Previous studies have predicted drug-perturbed GE using various approaches, including encoder–decoder architectures and generative adversarial networks (GANs) (Russkikh *et al.* 2020, Targonski *et al.* 2020, Hetzel *et al.* 2022, Wei *et al.* 2022, Qi *et al.* 2024). Encoder–decoder-based approaches effectively predict drug-perturbed GE using cell-line and chemical features, such as molecular graphs and SMILES representations (Hetzel *et al.* 2022, Qi *et al.* 2024). Specifically, PRnet achieved remarkable performance in predicting drug-perturbed GE using unperturbed GE and functional-class fingerprints as input features (Qi *et al.* 2024). However, these models fail to adequately capture gene–gene correlations owing to design failures in accounting for such covariation structures. Since gene–gene correlations reflect the biological pathways driving drug-induced cellular responses, models that fail to capture these correlations produce predictions with limited biological validity (Kim *et al.* 2021, Shu *et al.* 2021). GAN-based approaches employ generator networks with strong modeling capabilities to generate predictions (Russkikh *et al.* 2020, Targonski *et al.* 2020, Wei *et al.* 2022). Nevertheless, most studies do not fully incorporate perturbation-condition features, limiting any generalization. Furthermore, the capacity of these studies to capture gene–gene correlations remains limited.

Diffusion models have emerged as a promising approach to address these limitations (Luo *et al.* 2024, Zhang *et al.* 2024, Sefer 2025, He *et al.* 2026). Unlike encoder–decoder or GAN-based approaches, diffusion models learn the joint distribution of complex data by progressively denoising random noise through probabilistic sampling processes (Ho *et al.* 2020). Consequently, these models capture the covariance structure among genes, preserving gene–gene correlations. Diffusion models have also been applied to transcriptomic data for single-cell generation (Luo *et al.* 2024, He *et al.* 2026). These single-cell generative models primarily aim to characterize cellular heterogeneity or cell-state transitions. However, they do not address robust generalization to unseen drugs or the ability to model responses across a combinatorial space of continuous pharmacological variables including compound structure, treatment dose, and time. More directly relevant to GE prediction, PertDiT has demonstrated the feasibility of applying a diffusion model to predict drug-perturbed GE by incorporating transformer-based denoisers (Hu *et al.* 2026). PertDiT has also achieved state-of-the-art (SOTA) performance using unperturbed GE and drug text embedding vectors as condition features in the denoiser. However, since PertDiT performs the diffusion process directly in the high-dimensional GE space, this model incurs a high computational cost and is prone to training instability. Moreover, the denoiser in PertDiT predicts only the mean of the posterior, while fixing the variance to a constant value, thereby limiting the ability of the model to capture the full distributional structure of transcriptomic responses. Conversely, subsequent research on diffusion models has demonstrated that learning both the mean and variance generally improves the variational lower bound and facilitates log-likelihood optimization (Nichol and Dhariwal 2021). Log-likelihood serves as a key metric for evaluating generative model quality, and its optimization enables models to capture diverse modes of the data distribution more effectively (Razavi *et al.* 2019). This suggests that modeling both the mean and variance in diffusion model denoisers is crucial for capturing the distributional structure of real data and enhancing generation quality.

Among diffusion model variants, latent diffusion models (LDMs) offer particular advantages for high-dimensional data. LDMs perform diffusion in a compressed latent space rather than in the original data space, significantly reducing computational costs while maintaining generation quality (Rombach *et al.* 2022). This latent space representation enables more efficient learning of complex data distributions and provides improved control over the generation process. For the LINCS L1000, which involves approximately 1000 landmark genes with complex correlation structures, the dimensional reduction inherent in LDMs can help capture essential biological patterns while avoiding the computational burden of operating in the full GE space.

This study proposes an approach for predicting condition-aware drug-perturbed GE based on LDM. This approach encodes GE into a low-dimensional latent space using a variational autoencoder (VAE), enabling stable learning of complex GE patterns while improving computational efficiency. Furthermore, this model generates more robust and high-fidelity GE by predicting both the mean and variance of the posterior during the reverse diffusion process. To demonstrate the biological relevance of the generated GE, we predicted the half-maximal inhibitory concentration (IC_50_) and performed gene set enrichment analysis (GSEA). We also evaluated how the model reflects various perturbation conditions by visualizing latent representations using t-SNE.

Thus, this study aimed to (i) generate GE that faithfully reproduces the gene−gene correlation structure of real data, (ii) achieve high generation performance under unseen perturbation conditions, including novel cell lines and drugs that were absent from the training data, and (iii) ensure the predicted perturbed GE has biological relevance.

## 2 Methods

### 2.1 Model architecture

**Figure 1:**
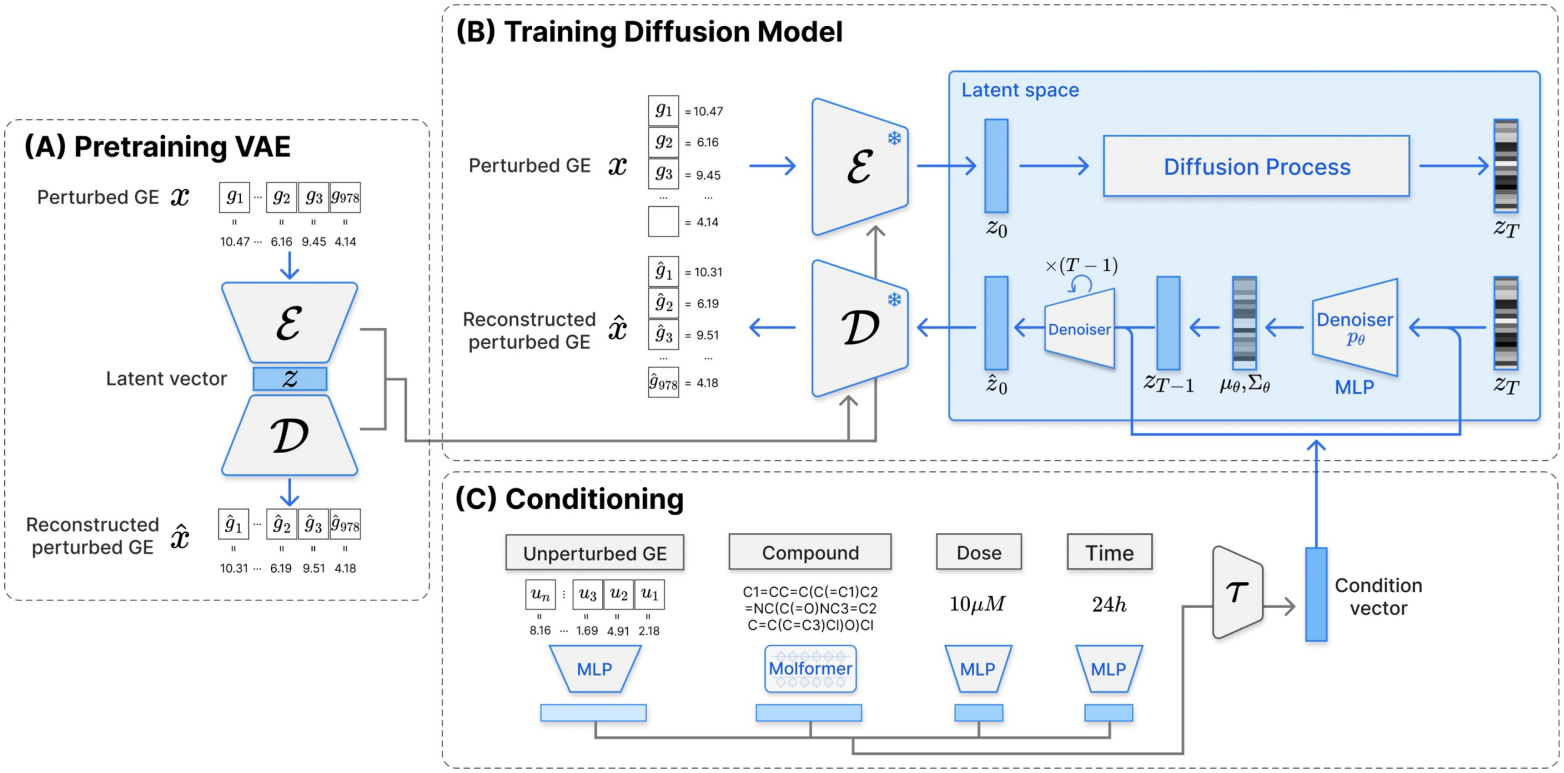
Overview of model architecture. (A) The VAE is pretrained on perturbed GE profiles. The encoder maps perturbed GE onto a latent space, from which the decoder can accurately reconstruct the original input. **(B)** The diffusion model is trained in the latent space provided by the pretrained VAE. The schematic illustrates both the forward and reverse diffusion processes, where the denoiser predicts the mean and variance of latent representations at each timestep. **(C)** Four conditioning features are incorporated in the reverse diffusion process: unperturbed GE, compound structure, treatment dose, and treatment time.

This study employs an LDM framework to generate condition-aware drug-perturbed GE (Fig. 1). The model comprises two main components: a VAE and a diffusion model. Initially, the VAE is pretrained on perturbed GE to obtain a stable latent representation (Fig. 1A). The pretrained VAE then maps the perturbed GE onto latent space, where the diffusion model performs training and inference (Fig. 1B). During the reverse diffusion process, the denoiser model predicts both the mean and variance of the latent distribution, thereby enabling more robust GE generation. Furthermore, diffusion models capture gene–gene correlations more comprehensively than deterministic models by learning to approximate complex multivariate distributions through an iterative denoising process. Finally, by integrating diverse perturbation conditions during training, the model learns condition-aware latent representations that enhance prediction performance for conditions absent from the training data (Fig. 1C).

### 2.2 Data preprocessing

This study used the LINCS L1000 dataset, which provides GE data from genetic (shRNA, cDNA) and chemical (small molecules) perturbations across various cell lines, compounds, time points, and dose conditions (Subramanian *et al.* 2017). The complete dataset contains experimentally measured GE for 978 landmark genes and imputed GE for 11,350 additional genes using a transformation matrix. To ensure data reliability, this study used only landmark gene data, available in three versions: phase I, phase II, and beta, each categorized into five levels based on the data processing pipeline. We used level 3 quantile-normalized GE profiles from phase I. The following data cleaning procedures were applied: (i) removal of compounds appearing fewer than 5 times in the entire datasets; (ii) removal of compounds with invalid SMILES strings that could not be successfully parsed by RDKit (2023); (iii) pairing of unperturbed and perturbed observations. The final dataset comprised 836,841 observations across 82 cell lines and 17,766 compounds, covering diverse treatment doses and time conditions.

### 2.3 VAE pretraining

**Figure 2:**
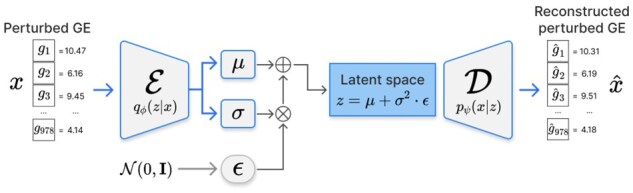
VAE structure. The encoder maps perturbed GE profiles onto a latent space parameterized by the mean and variance. The decoder reconstructs the original profiles from latent samples obtained via the reparameterization trick.

Perturbed GE is represented as a 978-dimensional vector; training a diffusion model directly on this high-dimensional space is computationally expensive and may lead to unstable optimization (Rombach *et al.* 2022). Therefore, we employed a VAE to map the GE profiles onto a lower-dimensional latent space, after which we trained the diffusion model (Fig. 2).

The VAE follows an encoder–decoder architecture (Kingma and Welling 2013). Given an input GE vector x, the encoder approximates the posterior distribution of the latent representation z, denoted as $q_{\phi }(z|x)$, by predicting its mean $\mu_{\mathrm{VAE}}$ and log-variance $\log\sigma_{\mathrm{VAE}}^{2}$. The decoder reconstructs the original GE profile from the latent representation by modeling the conditional distribution $p_{\psi }(x|z)$.

The VAE is trained by maximizing the evidence lower bound (ELBO) (Kingma and Welling 2013):


(1) $$\mathrm{ELBO}=E_{q_{\phi }(z|x)}[\log p_{\psi }(x|z)]-D_{\mathrm{KL}}(q_{\phi }(z|x)\|p(z),$$

Where p(z) is the prior distribution, assumed to be a standard Gaussian N(0, I).

In practice, the VAE loss function is as follows:


(2) $$\begin{array}{c} L_{\mathrm{VAE}}=\frac{1}{N}\sum_{i=1}^{N}{\left\|x_{i}-{\hat{x}}_{i}\right\|}_{2}^{2}\\ +k\cdot \frac{1}{N}\sum_{i=1}^{N}(\mu_{\mathrm{VAE},i}^{2}+\sigma_{\mathrm{VAE},i}^{2}-\log\sigma_{\mathrm{VAE},i}^{2}-1), \end{array}$$

Where the first term denotes the reconstruction loss, which is measured in terms of the mean squared error. N denotes the number of samples. The second term is the Kullback–Leibler divergence that regularizes the latent distribution toward the prior (Kullback and Leibler 1951). $\mu_{\mathrm{VAE},i}$ and $\sigma_{\mathrm{VAE},i}^{2}$ denote the mean and variance of latent representations of ith sample, respectively. k is the KL term weight, which is set to 1 in our experiments. The values for the hyperparameters are provided in Table S1, is available at *Bioinformatics* online.

### 2.4 Diffusion model training

GE follows a high-dimensional and complex distribution that reflects nonlinear interactions among numerous genes. Therefore, we adopted a diffusion model, which has been shown to outperform existing generative approaches in terms of generation quality and diversity (Ho *et al.* 2020). A diffusion model represents complex data distributions through a Markovian sequence of Gaussian-noise and denoising steps, enabling flexible and stable modeling of multimodal biological data. This probabilistic formulation can enhance the biological plausibility of the predicted GE by capturing stochastic variation inherent in the GE (Sadria and Layton 2025). The hyperparameters of diffusion model can be found in Table S2, is available at *Bioinformatics* online.

#### 2.4.1 Forward diffusion

Given the latent representation $z_{0}$ obtained from the pretrained VAE, Gaussian noise is gradually injected over T timesteps. At each timestep t, the noisy latent representation is sampled according to the following distribution:


(3) $$q(z_{t}|z_{t-1})=N(z_{t};\sqrt{1-\beta_{t}}z_{t-1},\;\beta_{t}\cdot I),$$

Where $\beta_{t}\in (0,1)$ is the noise scale parameter at t. We employed a linear noise scheduler:


(4) $$\beta_{t}=\beta_{\min}+\frac{t-1}{T-1}(\beta_{\max}-\beta_{\min}),$$

Where $\beta_{\min}$ and $\beta_{\max}$ specify the starting and ending noise levels of the linear schedule, set to $1\times 10^{-5}$ and 0.01, respectively.

Equivalently, the forward process can be expressed in closed form as:


(5) $$q(z_{t}|z_{0})=N(z_{t};\sqrt{{\bar{\alpha }}_{t}}z_{0},\;(1-{\overset{-}{\alpha }}_{t})\cdot I),$$

Where $\alpha_{t}=1-\beta_{t}$ and ${\bar{\alpha }}_{t}=\Pi_{s=1}^{t}\alpha_{s}$. This forward process generates a sequence of increasingly noisy latent representations that approaches a standard Gaussian distribution as t→T.

#### 2.4.2 Reverse diffusion

The reverse diffusion process progressively denoises the latent representations by modeling the reverse conditional distribution $q(z_{t-1}|z_{t}).$ Since this distribution is intractable without knowledge of the entire data distribution, a parameterized neural network is used to generate an approximation:


(6) $$p_{\theta }(z_{t-1}|z_{t},\;c)=N(\mu_{\theta }(z_{t},t,\;c),\Sigma_{\theta }(z_{t},t,c)),$$

Where the condition vector c encodes the basal GE, compound structure, treatment dose, and treatment time.

Basal GE profiles for each cell line are embedded using a multilayer perceptron (MLP). Compound features are extracted by embedding molecular SMILES strings with Molformer, a transformer-based molecular representation model with rotary position embeddings (Ross *et al.* 2022). Treatment dose and time are embedded via independently parameterized MLPs; notably, while dose effects have been extensively studied due to their strong influence on GE changes, treatment time, despite its biological importance, has received less attention. Nonetheless, since cellular metabolism and signaling pathways evolve temporally, treatment duration is a critical factor influencing GE (Canzler *et al.* 2020). Therefore, time features were explicitly incorporated. The resulting embeddings are concatenated and compressed into the condition vector c through the MLP τ.

The denoising network takes three inputs: the noisy latent variable $z_{t},\;$embedding vector for the timestep t, and the condition vector c. This network predicts both the mean $\mu_{\theta }$ and the interpolation vector $s\in {\left[0,1\right]}^{d}$ for log variance estimation, where d denotes the latent dimensionality. To ensure training stability, we avoided directly predicting the variance; instead, we adopted the interpolation method from the improved diffusion model approach, using s to compute the covariance matrix $\Sigma_{\theta }$ for $z_{t-1}$ as follows (Nichol and Dhariwal 2021):


(7) $$\Sigma_{\theta }(z_{t},\;t,c)=\exp(s\cdot \log(\beta_{t})+(1-s)\log{\tilde{\beta }}_{t}).$$

This interpolation occurs between an upper bound, the forward process variance $\beta_{t}I$, and a lower bound, the posterior variance ${\tilde{\beta }}_{t}I$, where $\tilde{\beta }=\frac{1-{\bar{\alpha }}_{t-1}}{1-{\bar{\alpha }}_{t}}\beta_{t}$. By constraining the predicted variance within this stable range, this technique prevents the numerical issues that arise when learning the variance directly.

At each reverse time step, $z_{t-1}$ is sampled from the predicted Gaussian distribution that is composed of $\mu_{\theta }$ and $\log\Sigma_{\theta }$. After T denoising steps, the final latent representation ${\hat{z}}_{0}$ is decoded by the pretrained VAE decoder to reconstruct the perturbed GE profile $\hat{x}$.

The diffusion model is trained to predict both the mean and the log-variance of the reverse distribution $p_{\theta }(z_{t-1}|z_{t},\;c)$. Given the true posterior from the forward process, we employed the negative log-likelihood of a Gaussian distribution. For a predicted Gaussian distribution with mean $\mu_{\theta }$ and log-variance $\log\Sigma_{\theta }$, the negative log-likelihood of the target $z_{t-1}$ is:


(8) $$L_{\mathrm{var}}=\frac{1}{2}E[\frac{1}{\Sigma_{\theta }}⊙{\left(z_{t-1}-\mu_{\theta }\right)}^{2}+\log\Sigma_{\theta }],$$

Where ⊙ denotes element-wise multiplication.

This objective can be interpreted as the sum of two complementary components. The first is a precision weighted mean squared error, which penalizes prediction errors more strongly when the model assigns low variance, thereby encouraging accurate reconstructions under confident predictions. The second is a variance regularization term, represented by $\log\Sigma_{\theta }$, which discourages collapse to near-zero variance, thereby preventing overconfident estimates.

The target $z_{t-1}$ is obtained from the forward process. Given $z_{0}$ and sampled noise $\epsilon_{t}∼N(0,\;I)$, we can compute $z_{t}$ and then derive the corresponding $z_{t-1}$ using the reparameterization:


(9) $$z_{t-1}=\frac{\sqrt{{\bar{\alpha }}_{t-1}}\beta_{t}}{1-{\bar{\alpha }}_{t}}z_{0}+\frac{\sqrt{\alpha_{t}}{(1-\bar{\alpha }}_{t-1})}{1-{\bar{\alpha }}_{t}}z_{t}+\sqrt{{\tilde{\beta }}_{t}}\epsilon_{t-1}.$$

The overall training objective is:


(10) $$L_{\mathrm{total}}=E_{t,z_{0},\epsilon }[L_{\mathrm{var}}(t)],$$

where timesteps are sampled uniformly, t∼U(1,T). This variational loss drives the model to learn accurate mean predictions, which are essential for stable, high-fidelity generation.

## 3 Results

### 3.1 Model performance and generalization ability

We first assessed the performance of the VAE, which serves as the foundation for constructing a biologically meaningful latent space (Supplementary Method 1.1, is available at *Bioinformatics* online). The VAE demonstrated highly stable reconstruction of perturbed GE profiles, achieving a Pearson correlation coefficient (PCC) of 0.957 ± 0.001 and an *R*^2^ score of 0.916 ± 0.001 between the input and reconstructed expression vectors. These results indicate that the VAE effectively captures the intrinsic structure of transcriptomic data. Consequently, the learned latent representation provides a robust basis for subsequent diffusion training, minimizing information loss and ensuring the reliability of generated GE from the diffusion model.

Using the latent representations learned by the VAE, we next evaluated the performance of the diffusion model in reconstructing perturbed GE profiles under unseen perturbation conditions (Supplementary Method 1.2, is available at *Bioinformatics* online). Each model was assessed using a 4-fold cross-validation. The proposed model consistently outperformed PRnet and PertDiT across both settings (Table 1). Under the unseen compound split, our model achieved a PCC of 0.870 ± 0.001 and an *R*^2^ score of 0.739 ± 0.001, significantly surpassing PertDiT, which yielded a PCC of 0.812 ± 0.009 and an *R*^2^ score of 0.637 ± 0.027. PRnet demonstrated lower performance with values of 0.782 ± 0.002 and 0.528 ± 0.013, respectively. Similarly, to assess generalization to novel cellular contexts, we implemented a cell-line-based partitioning strategy in which cell lines were assigned to disjoint subsets. Under the unseen cell line split, the proposed model maintained strong predictive accuracy, achieving a PCC of 0.743 ± 0.015 and an *R*^2^ score of 0.500 ± 0.033. This performance notably exceeded that of the baselines; PertDiT recorded values of 0.701 ± 0.007 and 0.428 ± 0.025, respectively, while PRnet showed lower accuracy with values of 0.692 ± 0.009 and 0.379 ± 0.026, respectively.

**Table 1:** GE reconstruction performance of baseline models and the proposed model.

Model	Unseen compound split		Unseen cell line split	
	PCC	R^2^ score	PCC	R^2^ score
PRnet	0.782 ± 0.002	0.528 ± 0.013	0.692 ± 0.009	0.379 ± 0.026
PertDiT	0.812 ± 0.009	0.637 ± 0.027	0.701 ± 0.007	0.428 ± 0.025
Proposed model	**0.870 ± 0.001**	**0.739 ± 0.001**	**0.743 ± 0.015**	**0.500 ± 0.033**

Bold values indicate the best performance.

Since the model takes molecular feature vectors derived from SMILES representations as input, the model can generate GE profiles for previously unseen compounds based on their chemical structures. Furthermore, the model demonstrated robust performance on unseen cell-line splits after integrating basal GEs as a cell-line-specific feature into the input. This allows the model to anchor its predictions on the initial biological state of a cell line. The model also incorporates drug treatment time and dose as conditioning features, and models both the mean and variance of the posterior distribution. Ablation studies confirmed that each conditioning feature and predicting both the mean and variance contribute meaningfully to performance, with their removal resulting in decreased PCC and *R*^2^ scores (Table S4, Supplementary Method 2, is available at *Bioinformatics* online). Consequently, incorporating molecular features, cellular contexts, treatment times, and doses, along with predicting both the mean and variance, enables our model to robustly predict drug-induced transcriptional responses across both previously unseen compounds and cell lines.

To further assess the generalizability of the proposed framework, we additionally validated the model on a single-cell perturbation dataset, Sci-plex3 (Srivatsan *et al.* 2020). The results demonstrate that the model maintains competitive performance at single-cell resolution, suggesting its broader applicability beyond bulk transcriptomic settings (Table S5, Supplementary Method 3, is available at *Bioinformatics* online).

### 3.2 Gene−gene correlation capturing

We evaluated how well each model preserved the gene–gene co-expression structure using two compounds with distinct mechanisms of action (Supplementary Method 4, is available at *Bioinformatics* online). For Trichostatin A, we examined the consistency of highly correlated genes by selecting the top 30 genes ranked by absolute correlation in the ground truth (GT) and measuring the overlap with the sets identified by each model. In total, 12 of the top 30 genes selected by the proposed model overlapped with the GT set, whereas PRnet and PertDiT showed no overlap (Table S6, is available at *Bioinformatics* online). Similar patterns were observed for GSK-1059615, in which the proposed model identified 12 overlapping genes, compared to 3 for both PRnet and PertDiT (Table S7, is available at *Bioinformatics* online). These results demonstrate that the proposed model more reliably recovers key co-regulated gene groups. To further assess the stability of this improvement across a broader range of compounds, we extended the analysis to all test compounds with sufficient observations (≥ 30 samples per compound, n = 1210). The proposed model achieved a mean overlap of 9.845 ± 4.243, compared to 4.583 ± 3.363 for PRnet and 5.367 ± 3.636 for PertDiT, demonstrating that the superior gene–gene correlation preservation is consistent across a wide range of compounds.

**Figure 3:**
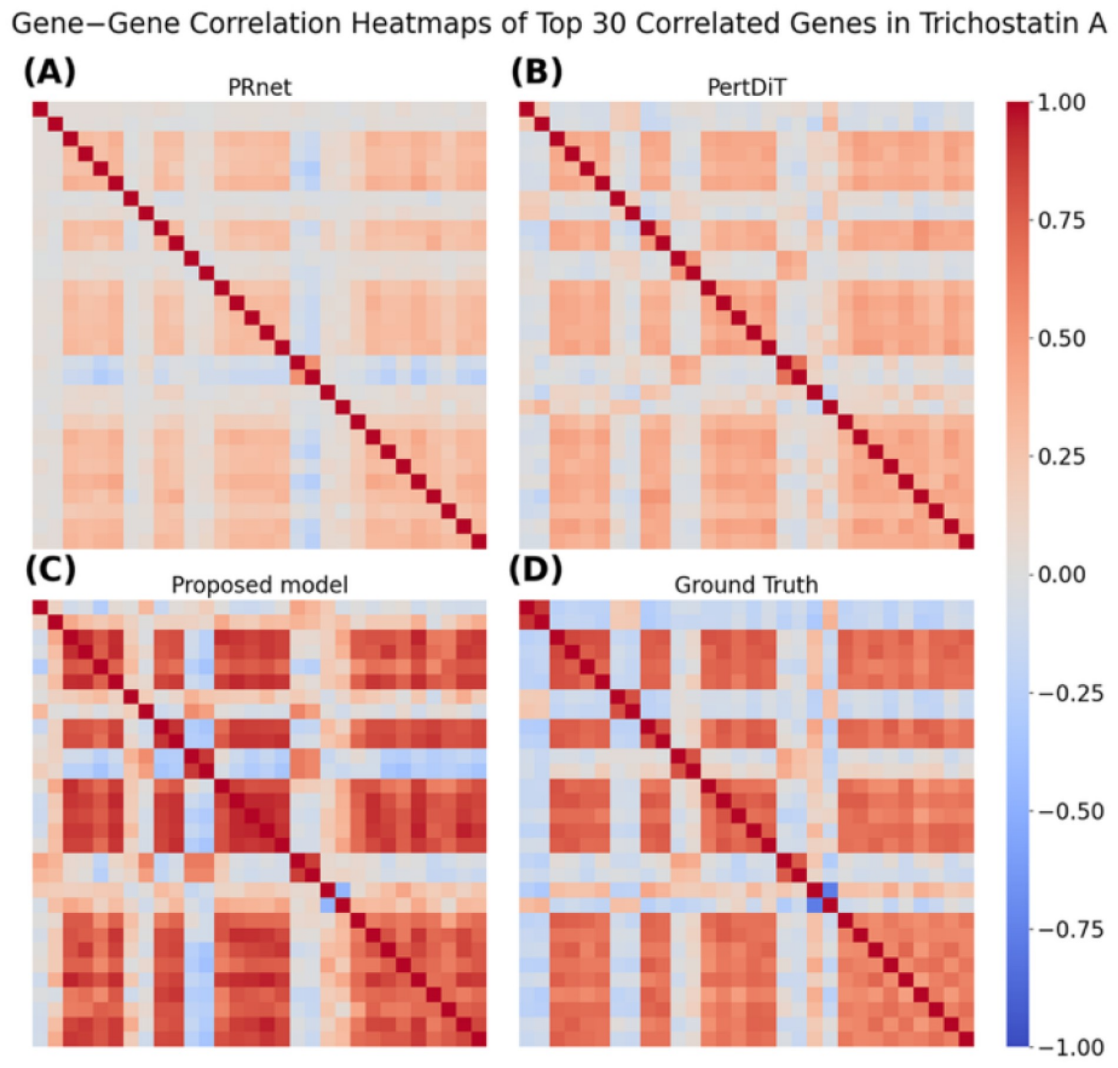
Gene−gene correlation heatmaps of the top 30 correlated genes in Trichostatin A. (A) PRnet, **(B)** PertDiT, **(C)** the proposed model, and **(D)** the GT. The top 30 genes were selected in GT, and pairwise correlation patterns among these genes were compared.

For the qualitative comparison, we visualized pairwise correlations among the top 30 genes from GT using heatmaps (Fig. 3, Fig. S1, is available at *Bioinformatics* online). The proposed model reproduced the major correlation patterns with substantially higher fidelity than the baseline models.

Overall, these results show that the proposed model improves reconstruction accuracy and more effectively preserves the gene–gene correlation structure, a key requirement for generating biologically meaningful transcriptomic profiles.

### 3.3 IC_50_ prediction using generated GE

We evaluated whether the GE profiles generated by the proposed model could improve downstream pharmacological predictions, specifically IC_50_ estimation (Supplementary Method 5, is available at *Bioinformatics* online). The IC_50_ regression model trained on the GE profiles generated by the proposed model achieved the best predictive performance, with an RMSE of 1.286 ± 0.037, a PCC of 0.908 ± 0.006, and an *R*^2^ score of 0.821 ± 0.010 (Table 2). In comparison, the models using profiles from PertDiT obtained values of 1.543 ± 0.069, 0.862 ± 0.013, and 0.742 ± 0.023, respectively. PRnet demonstrated reduced performance, with an RMSE of 1.580 ± 0.027, a PCC of 0.856 ± 0.006, and *R*^2^ score of 0.730 ± 0.009. This result indicates that our model preserves biologically relevant gene–gene correlations that influence drug sensitivity, thereby enhancing the predictive power of downstream pharmacological tasks (Ahmed *et al.* 2020).

**Table 2:** Comparison of IC_50_ prediction performance using perturbed GE profiles generated by baseline models and the proposed model.

Model	RMSE	PCC	R^2^ score
PRnet	1.580 ± 0.027	0.856 ± 0.006	0.730 ± 0.009
PertDiT	1.543 ± 0.069	0.862 ± 0.013	0.742 ± 0.023
Proposed model	**1.286 ± 0.037**	**0.908 ± 0.006**	**0.821 ± 0.010**

Bold values indicate the best performance.

Beyond these quantitative improvements, the ability to predict IC_50_ directly from computationally generated transcriptional responses underscores the potential of the model for virtual screening and drug repurposing. Unlike experimental perturbation assays, which are time-consuming and costly, our generative approach provides a scalable framework for estimating cellular responses across untested compound–cell line combinations. The generated GE profiles can serve as reliable substitutes for experimentally measured perturbation data, significantly reducing the time and cost required for large-scale drug evaluation. These findings demonstrate the potential of the proposed model as a practical tool for accelerating drug discovery and precision medicine.

### 3.4 Low-dimensional embedding for condition-dependent structure

**Figure 4:**
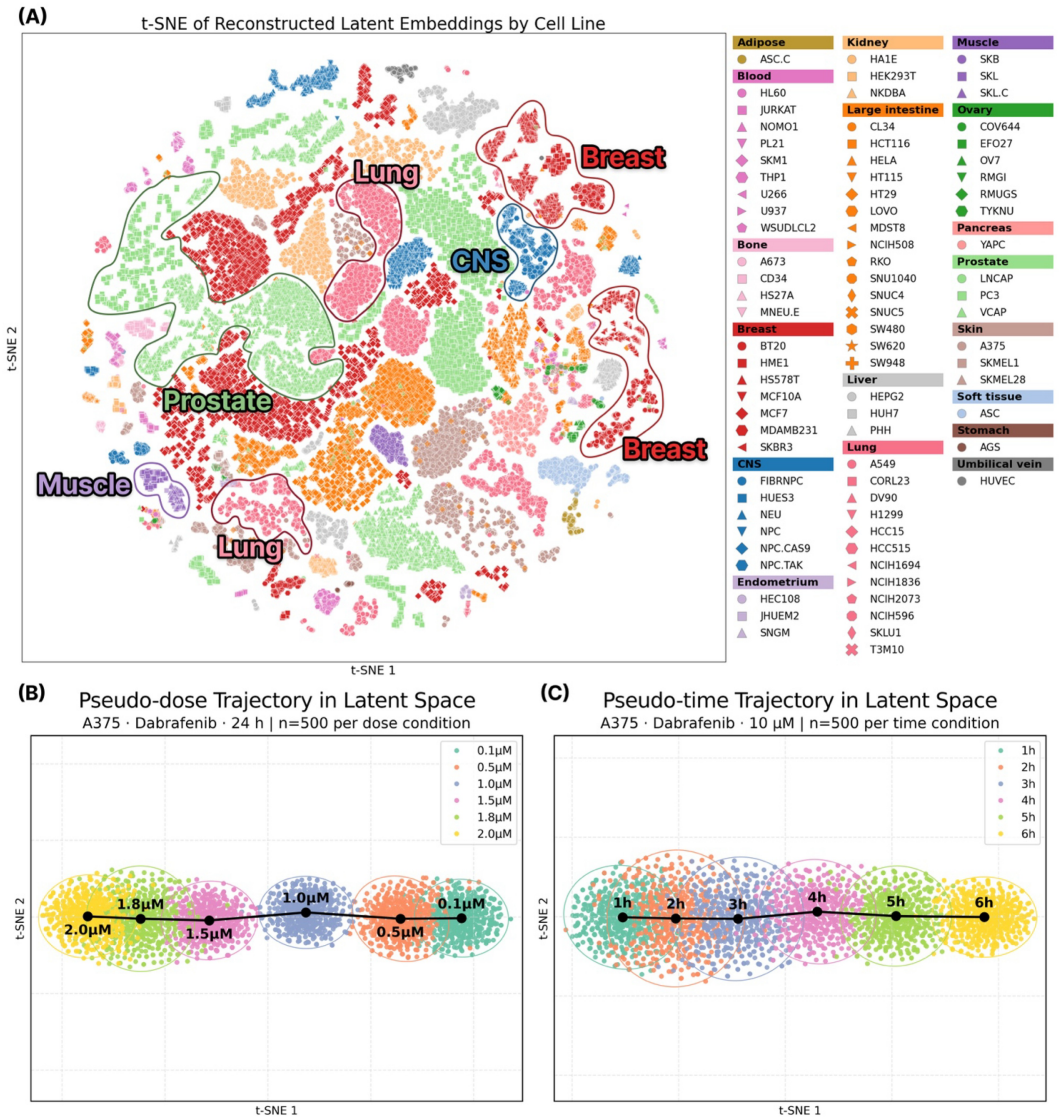
(A) Low-dimensional embedding of reconstructed latent representations. t-SNE visualization of reconstructed latent representations from the unseen compound test set. Samples form distinct clusters that correspond to organ lineages, with additional separation at the level of individual cell lines. **(B)** Pseudo-dose trajectory in the latent space for the A375–dabrafenib pair. For each dose level, 500 samples were generated at a fixed time point (24 h). **(C)** Pseudo-time trajectory in the latent space for the A375–dabrafenib pair. For each time level, 500 samples were generated at a fixed dose point (10 μM). The black dots in (B) and (C) are the centroids of each cluster.

To evaluate whether the diffusion model preserves perturbation–condition-dependent variation, we examined the reconstructed latent representation ${\hat{z}}_{0}$ using dimensionality reduction (Supplementary Method 6, is available at *Bioinformatics* online). We first assessed the extent to which cellular identity is maintained in the latent space (Fig. 4A). The t-SNE embedding displayed a clear organization by organ lineage, with distinct clusters corresponding to breast, central nervous system, prostate, lung, and muscle groups, followed by finer separation at the individual cell line level. These results indicate that the VAE latent space retains cell line-specific transcriptional structure, and that the subsequent diffusion process does not obscure these features.

We next analyzed how perturbation conditions, such as dose and time, are expressed within the latent space (Fig. 4B). Using the A375–dabrafenib pair, we generated samples under controlled dose and time conditions. When varying the dose at a fixed time, the centroids of the generated embeddings progressed smoothly from low (0.1 μM) to high (2.0 μM) dose. Likewise, varying the treatment time at a fixed dose (Fig. 4C) yielded a consistent ordering from early (1 h) to late (6 h) time points. In both cases, the centroids form well-aligned trajectories. This result indicates the model encodes dose and time as continuous directions in the latent space.

### 3.5 GSEA of the A375–vemurafenib

**Figure 5:**
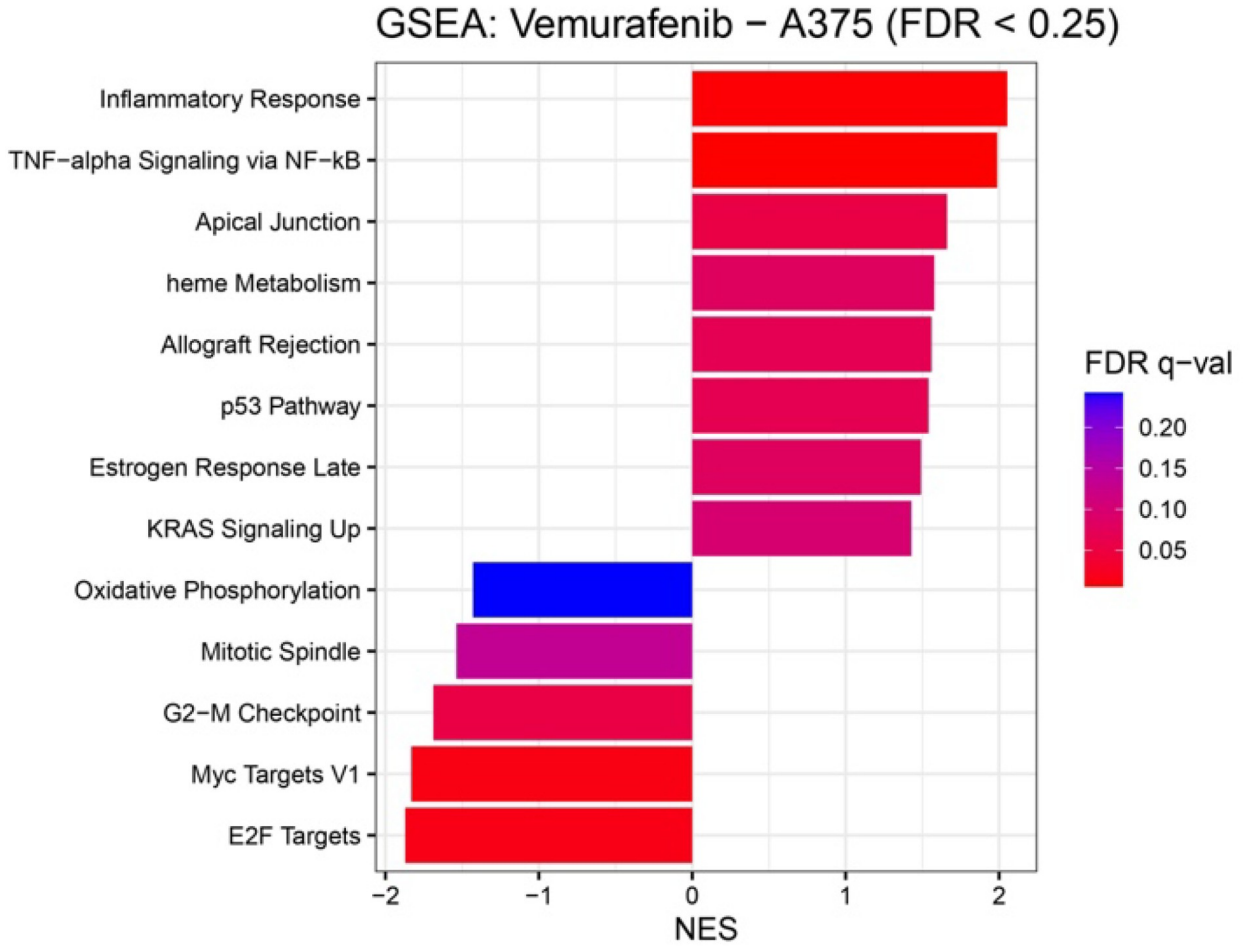
GSEA of generated transcriptional profiles for the A375–vemurafenib condition. Normalized enrichment score (NES) values for Hallmark gene sets with FDR q-values < 0.25. Positive NES values indicate pathways upregulated in the predicted vemurafenib response, including inflammatory and NF-κB-related pathways, whereas negative NES values correspond to downregulated cell-cycle-associated pathways. Bars are colored according to FDR q-values.

To examine whether the generated transcriptional profiles reflect the biologically established drug response, we performed GSEA using model-predicted GE for the A375–vemurafenib condition (Supplementary Method 7, is available at *Bioinformatics* online, Fig. 5). This pair was selected because A375 is a well-characterized melanoma cell line with the BRAF V600E mutation, which the vemurafenib directly targets (Bollag *et al.* 2010, Chapman *et al.* 2011, Al Hashmi *et al.* 2020). The molecular mechanism of action and downstream transcriptional consequences of vemurafenib have been extensively studied in BRAF-mutant melanomas, providing a well-defined reference for validating model-generated GE.

The enrichment patterns were highly concordant with known consequences of BRAF V600E inhibition in melanoma. The “inflammatory response” and “TNF-α via NF-κB” pathways were upregulated. A previous study has shown that vemurafenib induces senescence in BRAF V600E melanoma cells, leading to the release of cytokines (Peng *et al.* 2023). Moreover, pharmacological suppression of the MAPK pathway in BRAF-mutant melanomas enhances NF-κB activity and elevates TNF-α production (Xie *et al.* 2019). Several additional pathways exhibited positive enrichment, including the “p53 pathway,” “heme metabolism,” “estrogen response late,” and “apical junction.” Activation of the p53 pathway aligns with reports that intact p53 signaling enhances BRAF inhibition sensitivity (Ji *et al.* 2013, Krayem *et al.* 2016). Likewise, vemurafenib has been shown to induce heme oxygenase 1 expression in BRAF V600E melanoma cells, aligning with the observed enrichment of heme metabolism (Furfaro *et al.* 2020).

Notably, “estrogen response late” showed enrichment with GPER1, a Gs-coupled GPCR that elevates cAMP levels (Prossnitz and Barton 2011). BRAF inhibition triggers compensatory cAMP–PKA–CREB axis activation (Johannessen *et al.* 2013), and GPER signaling promotes differentiation and c-Myc degradation in BRAF-mutant melanoma (Natale *et al.* 2018). Consistent with this, we observed “Myc targets V1” downregulation, suggesting MAPK inhibition favors differentiation over proliferation (Dang *et al.* 2006).

Conversely, pathways associated with “cell-cycle progression” were markedly downregulated. Both “E2F targets” and “G2/M checkpoint” processes showed strong negative enrichment, consistent with the G1 arrest induced by vemurafenib in A375 cells and the subsequent repression of S-phase and mitotic gene pathways (Li *et al.* 2022). The suppression of “mitotic spindle” and “Myc targets V1” further supports this interpretation. MAPK pathway inhibition is known to reduce MYC abundance and suppress transcription of mitotic regulators, including those governing spindle assembly and Aurora B expression (Cui *et al.* 2010; Bonet *et al.* 2012).

Overall, the GSEA results demonstrate that the GE profiles generated by our model accurately capture the major biological processes underlying the response of BRAF-mutant melanoma to vemurafenib. The concordance with established inflammatory, metabolic, and cell-cycle regulatory changes supports the validity of the generated GE and indicates that the model captures drug-specific transcriptional pathways.

### 3.6 Computational cost evaluation in diffusion-based models

We compared the computational cost of the proposed model with PertDiT by measuring the FLOPs required for a single reverse diffusion step (Supplementary Method 8, is available at *Bioinformatics* online). The purpose was to quantify the reduction in computation achieved by denoising in a 256-dimensional latent space rather than in the original 978-dimensional GE space.

The results showed a substantial decrease in FLOPs for the proposed model. PertDiT required 401.979 M FLOPs per denoising step, whereas the proposed model required 1.217 M FLOPs under the same profiling conditions. This represents more than a 300-fold reduction in the number of operations necessary for a single reverse diffusion iteration.

This difference reflects the structural change introduced by the model design. PertDiT applies attention-based transformations directly to high-dimensional GE vectors, incurring a substantial computational burden. Conversely, the proposed model compresses GE profiles into a compact latent representation before diffusion, thereby reducing the dimensionality of all operations.

The reduction in FLOPs indicates that latent space diffusion offers a computationally efficient alternative to high-dimensional diffusion architectures, especially in multistep denoising or large-scale screening scenarios.

## 4 Discussion

This study developed an LDM framework for predicting condition-aware drug-perturbed GE profiles. By combining a VAE-derived latent space with a diffusion process that estimates both the mean and variance of the posterior, this model achieved higher reconstruction accuracy and stronger generalization. This model also more faithfully recovered gene–gene correlations, which is essential for producing biologically interpretable transcriptional responses.

Previous encoder–decoder and GAN-based models have shown utility in predicting transcriptomic changes, but their ability to capture co-expression patterns is limited. Conversely, diffusion models process the joint distribution of latent variables and preserve co-expression structure more effectively (Dhariwal and Nichol 2021; Xiao *et al.* 2021). The quantitative correlation analyses and the recovery of highly co-expressed genes support this conclusion. The use of a latent space provided a stable and computationally efficient setting for diffusion training, avoiding difficulties that arise when operating directly in high-dimensional expression space.

The performance improvements observed in the IC_50_ prediction task further indicate that biologically coherent GE profiles can enhance downstream pharmacological models. The proposed framework produced profiles that yielded more accurate IC_50_ estimates than those generated by PRnet or PertDiT. This underscores the practical advantage of models that preserve realistic gene–gene relationships rather than merely minimizing reconstruction error. The latent space structure also reflected differences across cell lines and responded continuously to changes in dose and time, which demonstrates that the model can represent graded perturbation effects.

Despite these strengths, several limitations remain. The model was trained on L1000 landmark genes, which represent only part of the transcriptome; extending the framework to whole-transcriptome measurements or other perturbation datasets would broaden applicability. The conditioning features included basal GE, molecular structure, dose, and time, although additional cellular information, such as chromatin state or pathway activity, was not incorporated. These factors may improve predictions when gene regulation is strongly condition-dependent. Although the denoiser is designed to predict both the mean and variance of the reverse diffusion posterior, the predicted variance converged to a near-constant value across conditions, precluding its direct use as a condition-aware confidence signal. This behavior likely reflects the population-averaged nature of bulk RNA-seq profiles, in which condition-induced transcriptional shifts are largely deterministic, thereby minimizing aleatoric uncertainty. The variance term nonetheless contributed to improved predictive performance, as confirmed by ablation study (Table S4, is available at *Bioinformatics* online). Developing more expressive variance parameterizations that yield condition-dependent uncertainty estimates remains an important direction for future work.

In summary, the LDM framework addressed several limitations of previous methods by providing predictions that better preserve transcriptomic structure and remain stable under unseen perturbation conditions. These results indicate that diffusion-based generative modeling can serve as a reliable foundation for future applications in perturbation modeling and pharmacogenomics.

## 5 Conclusions

This work introduced a latent diffusion model framework for predicting condition-aware drug-perturbed GE. The proposed approach achieved SOTA performance by combining a VAE for latent space construction with a diffusion process that jointly models the mean and variance. Our results demonstrate that the model reproduces the gene–gene correlation structure of real data more faithfully than prior methods, such as PRnet and PertDiT, and provides biologically consistent representations that enhance downstream applications, such as IC_50_ prediction.

These findings highlight the potential of diffusion-based generative models as versatile tools for drug discovery and precision medicine. By enabling more accurate modeling of cellular responses to chemical perturbations, the proposed framework may improve drug-sensitivity prediction, inform mechanism-of-action studies, and support the prioritization of candidate compounds. Future extensions that incorporate multimodal cellular features and provide broader experimental validation could further strengthen the translational impact of this approach. Exploring graph-based diffusion frameworks and explainability mechanisms over biological networks may offer additional directions for future work (Sefer and Kingsford 2016; Cetin and Sefer 2025).

## Supplementary Material

btag173_Supplementary_Data

## Data Availability

The L1000 was downloaded from the Gene Expression Omnibus with the accession number (GSE92742). The IC_50_ data were downloaded from the GDSC2 website (https://www.cancerrxgene.org/). All code and data for the manuscript are available on the Zenodo website (https://doi.org/10.5281/zenodo.18871024).
