# Supplementary material for: Predicting condition-aware drug-induced transcriptional responses via a latent diffusion model
Source: Bioinformatics. 2026 Apr 8;42(4):btag173. doi: 10.1093/bioinformatics/btag173 (PMC13107963; doi:10.1093/bioinformatics/btag173)
Supplement: btag173_Supplementary_Data [file btag173_supplementary_data.docx]

**Supplementary Information**

**Supplementary Method 1. Performance Evaluation**

**1.1. VAE Evaluation**

The performance of the VAE is critical for the stable and efficient training of the diffusion model. Since the decoder in the VAE reconstructs the final predicted GE profile $\hat{x}$ from the denoised latent representation $\hat{z}_{0}$, the reconstruction fidelity sets a practical upper bound on the overall predictive performance of our framework. Therefore, we evaluated the reconstruction quality of the VAE using the Pearson correlation coefficient (PCC) and R2 score between the input and reconstructed GE vectors.

We employed a compound-based split guided by the SMILES string of drugs to prevent overlap across the subsets. First, 20% of the dataset was reserved as a fixed test set. Then, four-fold cross-validation was performed on the remaining 80%, yielding a 6:2:2 split for training, validation, and test sets, respectively. Thus, the test set remained constant across all folds, while the 80% pool was iteratively divided for model training into distinct training and validation sets.

**1.2. Diffusion Model Evaluation**

The generation performance of the diffusion model was evaluated using root-mean-square error (RMSE), PCC, and R2 scores by comparing predicted GE profiles with the ground truth (GT), i.e., the perturbed GE profiles from LINCS L1000. This predictive accuracy was benchmarked against the existing baseline models, PRnet and PertDiT (Hu, et al., 2026; Qi, et al., 2024).

To evaluate the generalization ability of the model under unseen perturbation conditions, we adopted a compound-based data-splitting strategy consistent with that described in Section 2.5.1. The dataset was divided into training, validation, and test sets in a 6:2:2 ratio, using the SMILES strings of the drugs to ensure that no compound appeared in more than one subset. Furthermore, we implemented a cell-line-based data-splitting strategy, in which cell lines were partitioned into disjoint subsets. These evaluation protocols enable an objective assessment of the robustness and the capacity of the model to predict transcriptomic responses to previously unseen drugs and cell lines.

**Supplementary Method 2. Ablation Study on Perturbation Conditions**

To evaluate whether treatment time and dose features contribute to model performance, we conducted an ablation study. Using the unseen compound data split, we trained and evaluated model variants by excluding time and dose inputs, respectively. All other factors remained unchanged.

To further evaluate the contribution of variance prediction in the reverse diffusion process, we trained an additional model variant in which the denoiser predicts only the mean of the posterior distribution, with the variance fixed to a constant value. This variant was trained and evaluated under the same unseen compound data split, with all other factors remaining unchanged.

**Supplementary Method 3. Validation on Sci-plex3 Single-Cell Perturbation Dataset**

To assess the generalizability of the proposed model at single-cell resolution, we conducted additional validation on the Sci-plex3 dataset (GSM4150378; (Srivatsan, et al., 2020)). We used the preprocessed version of this dataset as provided by the PRnet (Qi, et al., 2024) repository to ensure a fair and consistent comparison with the PRnet baseline.

We evaluated the model under two data split strategies: unseen compound split and unseen pathway split, following the evaluation protocol of PRnet. Unlike PRnet, however, we used a consistent test set across all folds to ensure comparability across conditions. Unseen cell line split was not performed, as the dataset contains only three unique cell lines.

Model performance was evaluated using Pearson correlation coefficient (PCC) and R^2^ score in cov_compound, following the evaluation metrics adopted in PRnet. The R^2^ score in cov_compound evaluates the R^2^ score of the average gene expression perturbed by the same compounds within the same cell line, and was preferred over the standard R^2^ metric as the latter yielded negative values when applied to this dataset.

To further benchmark the proposed model against a foundation model-based approach, we additionally compared it with scGPT (Cui, et al., 2024) on the Sci-plex3 dataset. Since scGPT was originally designed for genetic perturbation prediction and does not natively support chemical perturbation tasks, we adapted its fine-tuning pipeline as follows. The gene perturbagen representation was replaced with drug-level molecular embeddings encoded using MolFormer, identical to the molecular encoder used in our proposed model, ensuring that both models received equivalent chemical representations as input. Dose and time conditions were embedded using separate MLP encoders and incorporated as additional input features, consistent with the conditioning scheme of our proposed model. The adapted model was fine-tuned for 10 epochs on the Sci-plex3 training set, with pretrained weights initialized from the published scGPT checkpoint.

**Supplementary Method 4. Gene–Gene Correlation Capturing Evaluation**

Since gene–gene interactions underlie regulatory networks and biological processes, an important aspect of transcriptomic modeling is the ability to reproduce the correlation structure among genes. To assess this capability, we compared the predicted correlation patterns from the proposed model and the baseline models against the GT, defined as the correlation structure observed in the perturbed GE data profiles from LINCS L1000.

To minimize confounding effects arising from aggregating heterogeneous perturbations across diverse compounds and cell lines, we focused our evaluation on a specific compound context within the test set derived from the unseen compound split. We selected two compounds representing distinct mechanisms of action: Trichostatin A and GSK-1059615. Trichostatin A is a potent histone deacetylase inhibitor that produces complex transcriptomic changes (Karantzali, et al., 2008; Yamashita, et al., 2003; Yoshida, et al., 1990). This offers an ideal scenario for assessing whether a model can capture broad regulatory dependencies. GSK-1059615, a PI3K/mTOR dual inhibitor that blocks the entire PI3K–AKT–mTOR signaling cascade and induces cell cycle arrest and apoptosis (Bei, et al., 2019; Wu, et al., 2022). As a dual inhibitor targeting multiple nodes in this pathway, GSK-1059615 triggers complex adaptive cellular responses involving both FOXO-regulated transcription, generating broad transcriptional changes suitable for evaluating gene–gene correlation recovery (Rodrik-Outmezguine, et al., 2011). Both compounds were among the most frequently represented in the test set, ensuring sufficiently large sample sizes for robust correlation estimation. Using subsets of samples treated with each compound, we calculated the pairwise gene–gene correlations for both the generated and GT profiles.

We identified the top 30 most strongly correlated genes, ranked by absolute correlation magnitude, using each model and GT. We then examined the overlap among the highly correlated gene sets.

For the qualitative assessment, we visualized the pairwise correlations among the top 30 genes in the GT correlation matrix as heatmaps, enabling visual inspection of local co-expression patterns captured by each model.

**Supplementary Method 5. IC_50_ Prediction using Generated GE**

To further explore the practical utility of our model in drug discovery, we evaluated whether the generated GE profiles could improve drug-sensitivity prediction, focusing on the IC_50_ as a representative pharmacological endpoint.

We obtained IC_50_ values corresponding to specific compound, cell line, dose, and time conditions from the GDSC2 database by downloading the fitted dose–response descriptions (Yang, et al., 2013). Meanwhile, duplicates were removed for conditions with multiple IC_50_ entries corresponding to the same compound, cell line, dose, and time. We retained only IC_50_ measurements for cell lines with available basal GE profiles in the LINCS L1000 dataset, resulting in a total of 3457 IC_50_ values. Perturbed GE profiles were generated for each condition using either baseline models or the proposed model.

To predict IC_50_ values, we constructed a downstream regression model, utilizing an MLP architecture. The input to this model consisted of a concatenated vector of the generated perturbed GE and the corresponding compound embeddings extracted by Molformer (Ross, et al., 2022). The perturbed GE profiles were normalized using a standard scaler fitted exclusively on the training data, which was then consistently applied to the validation and test sets to prevent data leakage. The IC_50_ prediction dataset was divided into training, validation, and test sets. First, 10% of the total data was selected as the test dataset. We then applied 5-fold cross-validation to the remaining 90% of the dataset. The regression model was trained by minimizing the mean squared error loss function. This setup allowed us to quantify the increase in predictive performance achieved when using GE profiles generated by our model compared to the baseline models. The hyperparameters for IC_50_ predictor are presented in Table S3.

**Supplementary Method 6. Low-dimensional Embedding for Condition-dependent Structure**

We performed dimensionality-reduction and visualization analyses to evaluate whether the generated GE profiles reflect variations in cell line identity, treatment dose, and treatment time. We first assessed whether the latent representations produced by the model preserve cell-line–specific features. For this purpose, we applied t-SNE to the reconstructed latent representations $\hat{z}_{0}$ of the test samples from the unseen compound split. Before t-SNE, we reduced the 256-dimensional latent vectors to 100 dimensions using principal component analysis (PCA), which is commonly used to denoise high-dimensional embeddings and improve the stability of subsequent t-SNE optimization (van der Maaten and Hinton, 2008).

To examine whether dose and time information were also reflected in the latent space, we generated pseudo-trajectories under controlled perturbation conditions. We selected the A375–dabrafenib pair as a representative example, among the exclusive cell line–compound pairs in the test set. This selection was motivated by the observation that dabrafenib induces distinct, graded transcriptional alterations by inhibiting the MAPK signaling pathway in BRAF V600E mutant melanoma cells (Pratilas, et al., 2009; Rheault, et al., 2013); thus, making the combination suitable for investigating whether the latent space captures graded responses to changes in treatment intensity. For each pair, we generated 500 samples per time point, holding the dose constant, and 500 samples per dose level, holding the time constant. This procedure yielded 500 $\times N_{\mathrm{time}}$ and 500 $\times N_{\mathrm{dose}}$ samples for the time-varying and dose-varying settings, respectively. PCA and t-SNE were applied to the resulting $\hat{z}_{0}$ values. For visualization, we computed the centroid of the t-SNE embeddings for each dose or time condition and connected these centroids in ascending order, thereby describing the progression of the latent representations along the respective condition axis.

**Supplementary Method 7. GSEA**

We performed GSEA to evaluate the biological validity of the generated GE profiles. We selected the vemurafenib–A375 pair from the test set of the unseen compound split for validation. Vemurafenib is a BRAF V600E inhibitor commonly used to treat melanoma, and A375 is a melanoma cell line with the BRAF V600E mutation (Al Hashmi, et al., 2020; Bollag, et al., 2010; Chapman, et al., 2011). A total of 54 predicted samples were extracted for this pair. To correct batch effects and maintain consistency with the LINCS L1000 processing pipeline from level 3 to level 4, we applied robust z-score normalization using plate-matched vehicle controls (Subramanian, et al., 2017) (Eqn. 11).

| $z_{i}=\frac{x_{i}-\mathrm{median}(C)}{1.4826\cdot\mathrm{MAD}(C)}$ | (3) |
| --- | --- |

where $x$ is the predicted GE value for a given gene, and $C$ is the vector of the control GE values for the same gene from the corresponding plate. A constant of 1.4826 represents the standard scaling factor that converts median absolute deviation (MAD) into a consistent estimator of the standard deviation.

A one-sample t-test was then applied to the normalized z-score vectors across the 54 samples, and genes were ranked in descending order of their t-statistic (Hung, 2013), which reflects both the average effect size and consistency across samples. The pre-ranked GSEA was performed using the GSEAPY Python package (Fang, et al., 2023).

In this study, enrichment was evaluated primarily using the Hallmark gene set collection from the Molecular Signatures Database (MSigDB) (Liberzon, et al., 2015). Hallmark gene sets comprise 50 curated sets that represent well-defined biological processes. These sets are constructed by integrating and filtering overlapping or noisy sets across multiple MSigDB collections to produce high-coherence signatures that capture the core expression patterns of major cellular programs. Hallmark gene sets are widely used for assessing global transcriptional responses due to their reduced redundancy and improved interpretability, and are appropriate for evaluating perturbation signatures. Following the standard GSEA significance criterion, pathways with a false discovery rate (FDR) q-value < 0.25 were considered significantly enriched and were used for downstream interpretation (Subramanian, et al., 2005).

**Supplementary Method 8. Computational Cost Evaluation in Diffusion-based Models**

To compare the computational requirements of diffusion-based architectures, we quantified the floating-point operations (FLOPs) required for a single reverse diffusion step in the proposed model and in PertDiT. FLOPs were computed using thop, a PyTorch-compatible profiling tool, by measuring the number of floating-point operations executed during one forward pass of each denoising network.

For PertDiT, FLOPs were evaluated in the original 978-dimensional GE space, consistent with its published configuration. For the proposed model, the diffusion process operates in a 256-dimensional latent space generated by the VAE, enabling direct assessment of the computational reduction achieved by latent-space diffusion.

All FLOPs measurements were obtained using an identical input structure to ensure comparability. A batch size of one was used for profiling, and dummy tensors were generated for each model input, including basal expression, drug representation, and diffusion time step.

**Supplementary Method 9. MC Dropout-Based Epistemic Uncertainty Estimation**

To assess whether the proposed model produces meaningful confidence signals alongside its transcriptional predictions, we implemented a Monte Carlo (MC) Dropout-based epistemic uncertainty estimation framework applied to the reverse diffusion process (Gal and Ghahramani, 2016).

During standard inference, the model operates in evaluation mode with all stochastic components disabled. For MC Dropout inference, we retained the evaluation mode for all layers except Dropout, which was kept in training mode to introduce stochastic variation across forward passes. This configuration fixes Batch Normalization statistics while allowing Dropout masks to vary at each forward pass, isolating parameter-level epistemic uncertainty from other sources of variation.

For each test sample, we performed $N=50$independent stochastic reverse diffusion trajectories. All trajectories were initialized from an identical Gaussian noise vector $z_{T}\mathcal{\sim N}(0, \mathbf{I})$ in the latent space, ensuring that observed variation across trajectories arises solely from Dropout stochasticity rather than differences in initialization. Each trajectory proceeded through $S=50$ uniformly spaced reverse diffusion steps over the full timestep range $[0,T-1]$.

At each reverse diffusion step $s$, the per-step epistemic variance was computed as the mean variance across the latent dimension over the $N$ stochastic predictions:

| $v_{s}=\frac{1}{d}\sum_{j=1}^{d} \mathrm{Var}_{n=1}^{N}[z_{s,n,j}]$ | (1) |
| --- | --- |

where $d$ denotes the latent dimensionality and $z_{s,n,j}$ is the $j$-th latent dimension of the $n$-th stochastic trajectory at step $s$. The total epistemic uncertainty for each sample was defined as the cumulative sum of per-step variances across all reverse diffusion steps:

| $U=\sum_{s=1}^{S} v_{s}.$ | (2) |
| --- | --- |

To examine the relationship between data abundance and model uncertainty, we aggregated the total epistemic uncertainty $U$ by cell line. For each cell line, we computed the mean uncertainty across all test samples and correlated this with the number of training samples associated with that cell line using the Spearman rank correlation coefficient. To verify that the observed correlation was not driven by a small number of high-leverage cell lines, we repeated the analysis after excluding the top 10 cell lines by sample count.

**Supplementary Result 1. Epistemic Uncertainty Reflects Data Abundance Across Cell Lines**

To evaluate whether the epistemic uncertainty estimated via MC Dropout functions as a meaningful confidence signal, we examined its relationship with cell line-level data abundance in the training set (Supplementary Method 9).

As shown in Fig. S2, cell lines with fewer training samples consistently exhibited higher mean epistemic uncertainty, while data-rich cell lines yielded lower uncertainty estimates. This negative relationship was statistically robust, with a Spearman correlation of $\rho=-0.591 (p=5.16\times{10}^{-9}, n=82$cell lines). To confirm that this result was not driven by a small number of well-represented cell lines such as MCF7, VCAP, and PC3, we repeated the analysis after excluding the top 10 cell lines by sample count. The correlation remained statistically significant ($\rho=-0.418, p=2.62\times{10}^{-4}$), confirming that the pattern is not solely attributable to a small number of high-leverage data points. However, the reduction in effect size indicates that data-rich cell lines contribute meaningfully to the overall trend.

These results demonstrate that the epistemic uncertainty estimated by the model is sensitive to the degree of cellular context representation in the training data. Cell lines that were underrepresented during training are associated with higher predictive uncertainty, which is consistent with the theoretical interpretation of epistemic uncertainty as arising from insufficient training data coverage (Kendall and Gal, 2017). This property suggests that the model's uncertainty estimates can provide practical guidance in drug discovery applications, for example by flagging predictions for data-sparse cell lines as candidates for prioritized experimental validation.

**
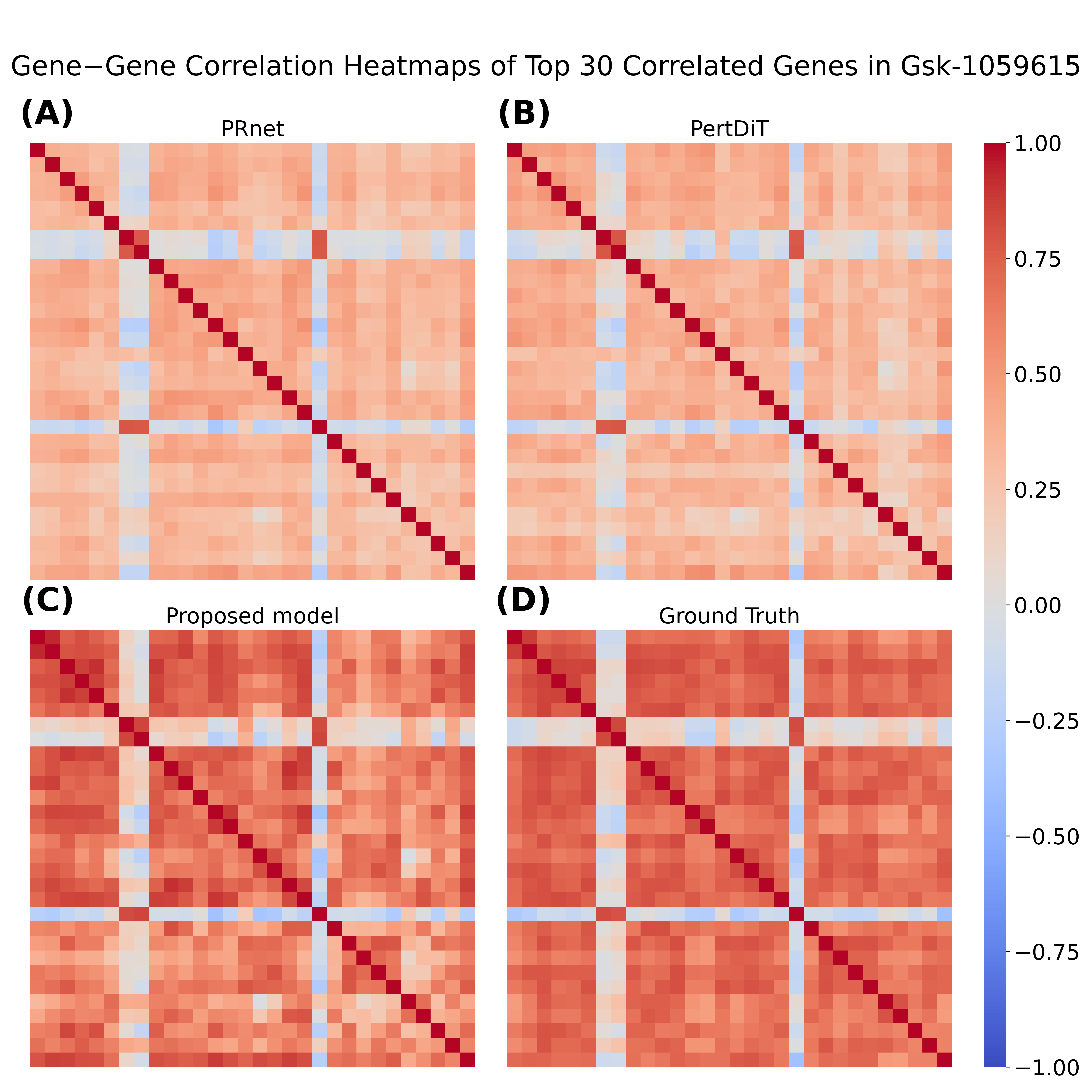
**

**Fig. S1.** Gene−gene correlation heatmaps of the top 30 correlated genes in GSK-1059615. Heatmaps are shown for (A) PRnet, (B) PertDiT, (C) the proposed model, and (D) the GT. The top 30 genes were selected in GT, and pairwise correlation patterns among these genes were compared.


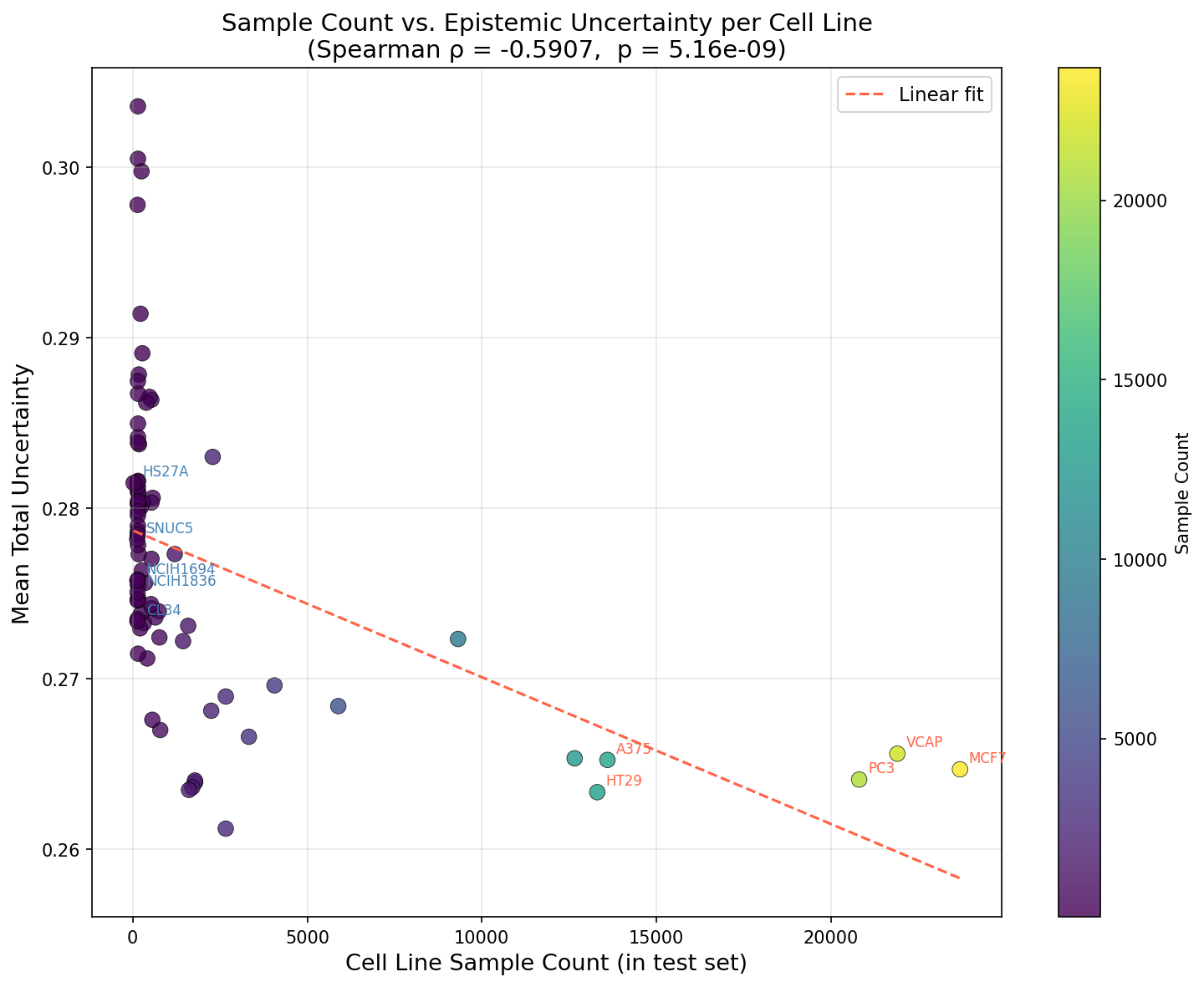


Fig. S2. Relationship between cell line training data abundance and MC Dropout epistemic uncertainty. Scatter plot showing the mean total epistemic uncertainty per cell line as a function of the number of training samples for that cell line in the test set ($\boldsymbol{n=82}$ cell lines). Each point represents one cell line, colored by sample count. The dashed line indicates the linear regression fit. Spearman $\boldsymbol{\rho=-0.591, p=5.16\times}\boldsymbol{10}^{\boldsymbol{-9}}$. Representative cell lines with high sample counts and low uncertainty, and cell lines with low sample counts and high uncertainty, are labeled. Epistemic uncertainty was estimated via MC Dropout inference using $\boldsymbol{N=}\boldsymbol{5}\boldsymbol{0}$ stochastic forward passes through the reverse diffusion process.

Table S1. Hyperparameters set of VAE.

| **Module** | **Hyperparameter** | **Value** |
| --- | --- | --- |
| Variational autoencoder | Input dimension | 978 or 5000  * 978: LINCS L1000, 5000: Sci-plex3 |
|  | Latent dimension | 256 |
|  | Encoder | Input → 512 → 256 → 256 |
|  | $\mu$/$\log\sigma$ projection head | 256 → 256 |
|  | Decoder | 256 → 512 (+ RB) → 512 (+ RB) → 256 → 512 → Input  * RB: Residual Block |
|  | Batch size | 1024 |
|  | KLD weight | 1 or 0.3  * 1: LINCS L1000, 0.3: Sci-plex3 |
|  | Optimizer | Adam |
|  | Scheduler | ExponentialLR ($\gamma$=0.99, 0.995)  * 0.99: LINCS L1000, 0.995: Sci-plex3 |
|  | Learning rate | 0.001, 0.0005  * 0.001: LINCS L1000, 0.0005: Sci-plex3 |
|  | Early stop patience | 20 |

Table S2. Hyperparameters set of LDM.

| **Module** | **Hyperparameter** | **Value** |
| --- | --- | --- |
| Latent diffusion model | Timesteps | 1000 |
|  | $\beta$ schedule | Linear [10^-5^, 0.01] |
|  | Sampling steps | 50 |
|  | Latent dimension | 256 |
|  | Basal GE encoder | 978 or 5000 → 512 → 512 → 256 → 256  * 978: LINCS L1000, 5000: Sci-plex3 |
|  | Compound encoder | 768 → 512 → 256 → 256 |
|  | Dose/time encoder | 1 → 32 |
|  | Condition encoder $\tau$ | (256 + 256 + 32 + 32) → 512 →256 →256 |
|  | Diffusion time $t$ embedding | 1 → 128 → 128 |
|  | Denoiser | (256 + 256 + 128) → 512 → 512 → 512 → 512  * 256: $z_{t}$, 256: condition vector, 128: diffusion $t$ embedding |
|  | Optimizer | Adam |
|  | Batch size | 256 * 2 GPUs (PyTorch Distributed Data Parallel) |
|  | Scheduler | NoamLR |
|  | Learning rate | 10^-4^ → 10^-3^ → 10^-4^ |
|  | Warmup epochs | 2 |
|  | Early stop patience | 30 |

Table S3. Hyperparameters set of IC_50_ predictor.

| **Module** | **Hyperparameter** | **Value** |
| --- | --- | --- |
| IC_50_ predictor | GE encoder | 978 or 5000 → 512 → 256 → 256  *978: LINCS L1000, 5000: Sci-plex3 |
|  | Compound encoder | 768 → 512 → 256 → 256 |
|  | Predictor | 512 → 256 → 256 → 128 → 1 |
|  | Optimizer | Adam |
|  | Learning rate | 5 × 10^-4^ |
|  | Weight decay | 1 × 10^-3^ |
|  | Scheduler | ReduceLROnPlateau |
|  | Scheduler mode | Min |
|  | Scheduler factor | 0.5 |
|  | Scheduler patience | 15 |
|  | Loss function | MSE (Mean Squared Error) |
|  | Batch size | 128 |
|  | Epochs | 300 |
|  | Early stop patience | 50 |

**Table S4.** Performance in ablation studies.

| **Model** | **PCC** | **R^2^ score** |
| --- | --- | --- |
| Proposed model w/o basal GE | 0.733 ± 0.001 | 0.487 ± 0.004 |
| Proposed model w/o compound | 0.865 ± 0.001 | 0.728 ± 0.002 |
| Proposed model w/o time | 0.861 ± 0.009 | 0.723 ± 0.016 |
| Proposed model w/o dose | 0.862 ± 0.006 | 0.723 ± 0.011 |
| Proposed model w/o variance (mean-only) | 0.809 ± 0.004 | 0.630 ± 0.007 |
| Proposed model | **0.870 ± 0.001** | **0.739 ± 0.001** |

Removal of features resulted in decreased performance, confirming their contribution to accurate modeling of perturbation-specific transcriptional response.

Table S5. GE reconstruction performance of baseline models and the proposed model in Sci-plex3.

| **Model** | **Unseen compound split** | | **Unseen pathway split** | |
| --- | --- | --- | --- | --- |
|  | **PCC** | **R^2^ score in cov_compounds** | **PCC** | **R^2^ score in cov_compounds** |
| PRnet | 0.430 ± 0.003 | 0.954 ± 0.004 | 0.435 ± 0.001 | 0.956 ± 0.005 |
| PertDiT | 0.474 ± 0.003 | **0.971 ± 0.004** | 0.484 ± 0.007 | **0.976 ± 0.002** |
| scGPT | 0.580 ± 0.000 | 0.793 ± 0.014 | 0.585 ± 0.000 | 0.794 ± 0.010 |
| Proposed model | **0.746 ± 0.000** | 0.969 ± 0.001 | **0.751 ± 0.000** | 0.973 ± 0.001 |

**Table S6.** Top 30 highly correlated genes identified by each model for Trichostatin A perturbation.

| **Rank in model** | **Proposed model** | **GT rank** | **PRnet** | **GT rank** | **PertDiT** | **GT rank** |
| --- | --- | --- | --- | --- | --- | --- |
| 1 | **PLEKHM1** | **26** | GATA3 | 453 | APPBP2 | 39 |
| 2 | SUPV3L1 | 70 | TBX2 | 454 | GATA3 | 453 |
| 3 | **CD40** | **16** | APPBP2 | 39 | TBX2 | 454 |
| 4 | **ARNT2** | **29** | NCOA3 | 40 | NCOA3 | 40 |
| 5 | RTN2 | 371 | NRAS | 129 | MYL9 | 121 |
| 6 | FAM69A | 38 | ST7 | 562 | COL1A1 | 141 |
| 7 | TESK1 | 34 | GATA2 | 205 | ST7 | 562 |
| 8 | **DNMT3A** | **15** | COL1A1 | 141 | GATA2 | 205 |
| 9 | PPOX | 69 | SERPINE1 | 287 | SERPINE1 | 287 |
| 10 | **GRB7** | **24** | BAMBI | 412 | HSPB1 | 120 |
| 11 | SNX13 | 36 | MYL9 | 121 | DUSP4 | 92 |
| 12 | GATA3 | 453 | RAD51C | 460 | FOSL1 | 89 |
| 13 | TBX2 | 454 | SH3BP5 | 457 | XBP1 | 259 |
| 14 | **PAPD7** | **3** | FOSL1 | 89 | BAMBI | 412 |
| 15 | IPO13 | 97 | PPARG | 90 | FHL2 | 131 |
| 16 | PLA2G4A | 72 | ECD | 223 | PPARG | 90 |
| 17 | CAMSAP2 | 233 | ANXA7 | 47 | CDK19 | 313 |
| 18 | **SHB** | **6** | HSPB1 | 120 | DDB2 | 178 |
| 19 | NVL | 63 | FHL2 | 131 | P4HA2 | 180 |
| 20 | **IFNAR1** | **14** | ST6GALNAC2 | 796 | NRAS | 129 |
| 21 | CASP10 | 55 | DUSP4 | 92 | ANKRD10 | 520 |
| 22 | FKBP4 | 32 | P4HA2 | 180 | PRSS23 | 130 |
| 23 | **ZNF395** | **4** | CDK19 | 313 | BAG3 | 307 |
| 24 | ST6GALNAC2 | 796 | CTSD | 309 | RAD51C | 460 |
| 25 | UBE2J1 | 81 | DDB2 | 178 | MYC | 296 |
| 26 | **FUT1** | **23** | KIF5C | 271 | PCBD1 | 187 |
| 27 | **PTPRF** | **17** | ANKRD10 | 520 | RGS2 | 542 |
| 28 | **BUB1B** | **10** | CGRRF1 | 352 | CTSD | 309 |
| 29 | WDR7 | 344 | XBP1 | 259 | IER3 | 238 |
| 30 | PPP1R13B | 268 | SPDEF | 355 | S100A13 | 226 |

For each model, the 30 genes with the highest absolute correlation coefficients were selected and ranked. The corresponding rank of each gene in the ground truth (GT) correlation structure is shown. Bold text indicates genes that also ranked within the top 30 in GT. The proposed model recovered 12 of the GT top 30 genes, whereas PRnet and PertDiT showed no overlap.

**Table S7.** Top 30 highly correlated genes identified by each model for GSK-1059615 perturbation.

| **Rank in model** | **Proposed model** | **GT rank** | **PRnet** | **GT rank** | **PertDiT** | **GT rank** |
| --- | --- | --- | --- | --- | --- | --- |
| 1 | **FKBP4** | **1** | MYL9 | 74 | **BIRC5** | **8** |
| 2 | **ARNT2** | **2** | COL1A1 | 75 | **CDK1** | **20** |
| 3 | SNCA | 408 | CALU | 66 | **TOP2A** | **7** |
| 4 | KIF2C | 409 | TRAM2 | 67 | MYL9 | 74 |
| 5 | **RFNG** | **30** | SERPINE1 | 96 | COL1A1 | 75 |
| 6 | FAM69A | 100 | **TOP2A** | **7** | COL4A1 | 93 |
| 7 | **TESK1** | **13** | **CDK1** | **20** | BNIP3L | 145 |
| 8 | **PAPD7** | **10** | P4HA2 | 187 | CALU | 66 |
| 9 | **SHB** | **18** | LIG1 | 40 | TRAM2 | 67 |
| 10 | **IFNAR1** | **3** | **BIRC5** | **8** | CCNB2 | 70 |
| 11 | **CD40** | **5** | COL4A1 | 93 | LIG1 | 40 |
| 12 | **BIRC5** | **8** | UBE2C | 84 | PLK1 | 63 |
| 13 | PLK1 | 63 | LOXL1 | 195 | SERPINE1 | 96 |
| 14 | PPOX | 32 | EZH2 | 41 | P4HA2 | 187 |
| 15 | CCNB2 | 70 | CCNA2 | 47 | EZH2 | 41 |
| 16 | NUSAP1 | 73 | SOX4 | 220 | CCNA2 | 47 |
| 17 | **PLEKHM1** | **19** | KIF5C | 153 | MELK | 120 |
| 18 | RTN2 | 50 | CCNB2 | 70 | MCM3 | 72 |
| 19 | CASP10 | 230 | CRTAP | 202 | CDC20 | 62 |
| 20 | **ORC1** | **9** | BNIP3 | 203 | LRP10 | 58 |
| 21 | SUPV3L1 | 55 | LRP10 | 58 | MVP | 83 |
| 22 | EXT1 | 52 | HSPB1 | 90 | KIF20A | 49 |
| 23 | VPS28 | 526 | HMGCR | 200 | SMC4 | 87 |
| 24 | CHAC1 | 253 | SOX2 | 278 | SFN | 265 |
| 25 | CDC20 | 62 | CDKN1A | 135 | TSTA3 | 266 |
| 26 | **CAMSAP2** | **14** | BNIP3L | 145 | DDR1 | 103 |
| 27 | UBE2J1 | 88 | MCM3 | 72 | PIK3R3 | 104 |
| 28 | CTNND1 | 43 | CDK5R1 | 154 | FKBP14 | 424 |
| 29 | ATP11B | 54 | CTSL | 110 | LOXL1 | 195 |
| 30 | MELK | 120 | TSC22D3 | 488 | HMGCR | 200 |

For each model, the 30 genes with the highest absolute correlation coefficients were selected and ranked. The corresponding rank of each gene in the GT correlation structure is shown. Bold text indicates genes that also ranked within the top 30 in GT. The proposed model recovered 12 of the GT top**-**30 genes, whereas PRnet and PertDiT showed 3 overlaps.

**References**

Al Hashmi, M., et al. Differential responsiveness to BRAF inhibitors of melanoma cell lines BRAF V600E-mutated. J Transl Med 2020;18(1).

Bei, S.H., et al. Inhibition of gastric cancer cell growth by a PI3K-mTOR dual inhibitor GSK1059615. Biochem Bioph Res Co 2019;511(1):13–20.

Bollag, G., et al. Clinical efficacy of a RAF inhibitor needs broad target blockade in BRAF-mutant melanoma. Nature 2010;467(7315):596–599.

Chapman, P.B., et al. Improved Survival with Vemurafenib in Melanoma with BRAF V600E Mutation. New Engl J Med 2011;364(26):2507–2516.

Cui, H., et al. scGPT: toward building a foundation model for single-cell multi-omics using generative AI. Nat Methods 2024;21(8):1470–1480.

Fang, Z.Q., Liu, X.Y. and Peltz, G. GSEApy: a comprehensive package for performing gene set enrichment analysis in Python. Bioinformatics 2023;39(1).

Gal, Y. and Ghahramani, Z. Dropout as a Bayesian Approximation: Representing Model Uncertainty in Deep Learning. Pr Mach Learn Res 2016;48.

Hu, Q., Chen, Z. and Gu, J. Predicting drug‐perturbed transcriptional responses using multi‐conditional diffusion transformer. Quantitative Biology 2026;14(1):e70016.

Hung, J.H. Gene Set/Pathway enrichment analysis. Methods Mol Biol 2013;939:201–213.

Karantzali, E., et al. Histone deacetylase inhibition accelerates the early events of stem cell differentiation: transcriptomic and epigenetic analysis. Genome Biol 2008;9(4):R65.

Kendall, A. and Gal, Y. What uncertainties do we need in bayesian deep learning for computer vision? Advances in neural information processing systems 2017;30.

Liberzon, A., et al. The Molecular Signatures Database Hallmark Gene Set Collection. Cell Syst 2015;1(6):417–425.

Pratilas, C.A., et al. (V600E)BRAF is associated with disabled feedback inhibition of RAF-MEK signaling and elevated transcriptional output of the pathway. Proc Natl Acad Sci U S A 2009;106(11):4519–4524.

Qi, X., et al. Predicting transcriptional responses to novel chemical perturbations using deep generative model for drug discovery. Nature Communications 2024;15(1):9256.

Rheault, T.R., et al. Discovery of Dabrafenib: A Selective Inhibitor of Raf Kinases with Antitumor Activity against B-Raf-Driven Tumors. Acs Med Chem Lett 2013;4(3):358–362.

Rodrik-Outmezguine, V.S., et al. mTOR Kinase Inhibition Causes Feedback-Dependent Biphasic Regulation of AKT Signaling. Cancer Discov 2011;1(3):248–259.

Ross, J., et al. Large-Scale Chemical Language Representations Capture Molecular Structure and Properties. 2022.

Srivatsan, S.R., et al. Massively multiplex chemical transcriptomics at single-cell resolution. Science 2020;367(6473):45–51.

Subramanian, A., et al. A Next Generation Connectivity Map: L1000 Platform and the First 1,000,000 Profiles. Cell 2017;171(6):1437–1452.e1417.

Subramanian, A., et al. Gene set enrichment analysis: A knowledge-based approach for interpreting genome-wide expression profiles. P Natl Acad Sci USA 2005;102(43):15545–15550.

van der Maaten, L. and Hinton, G. Visualizing Data using t-SNE. J Mach Learn Res 2008;9:2579–2605.

Wu, X.B., et al. Recent Advances in Dual PI3K/mTOR Inhibitors for Tumour Treatment. Front Pharmacol 2022;13.

Yamashita, Y., et al. Histone, deacetylase inhibitor trichostatin a induces cell-cycle arrest/apoptosis and hepatocyte differentiation in human hepatoma cells. Int J Cancer 2003;103(5):572–576.

Yang, W., et al. Genomics of Drug Sensitivity in Cancer (GDSC): a resource for therapeutic biomarker discovery in cancer cells. Nucleic Acids Res 2013;41(Database issue):D955–961.

Yoshida, M., et al. Potent and specific inhibition of mammalian histone deacetylase both in vivo and in vitro by trichostatin A. J Biol Chem 1990;265(28):17174–17179.
